# Congenital Myasthenic Syndrome Associated With SLC25A1 Gene Variant: The First Reported Case in Saudi Arabia

**DOI:** 10.7759/cureus.35808

**Published:** 2023-03-06

**Authors:** Ali Yahya B Alzahrani, Linah Saleh Abbas Alghamdi, Hanin Abdullah M Alghamdi, Ahmed Fahmy Hassan, Matar Ahmed Alsehemi

**Affiliations:** 1 Pediatrics, King Fahad Hospital, Al Baha, SAU; 2 Pediatric Critical Care Medicine, King Fahad Hospital, Al Baha, SAU; 3 Pediatric Neurology, King Fahad Hospital, Al Baha, SAU

**Keywords:** hypotonia, ophthalmoplegia, apnea, neuromuscular disorder, slc25a1 gene, congenital myasthenic syndrome

## Abstract

We report the case of a two-year-old full-term girl of consanguineous Saudi parents, who had a history of poor sucking, hypotonia, and bilateral ptosis, as well as recurrent pediatric intensive care unit (PICU) admissions with apnea and global developmental delay and unremarkable family history. A genetic study was conducted and whole exome sequencing (WES) identified a likely pathogenic homozygous variant c.842C>T p.(Ala281Val) in the SLC25A1 gene. This finding is consistent with the genetic diagnosis of autosomal recessive combined D-2- and L-2-hydroxyglutaric aciduria (D/L-2-HGA). Genetic testing results suggested a diagnosis of congenital myasthenic syndrome (CMS) type 23 [Online Mendelian Inheritance in Man (OMIM) #618197]. CMS is a highly heterogeneous group of neuromuscular junction (NMJ) disorders clinically and genetically and compromises the safety margin required for reliable neuromuscular transmission. Fortunately, we suspected a CMS in our patient, and the initiation of management with pyridostigmine has substantially improved the patient's condition.

## Introduction

Congenital myasthenic syndrome (CMS) is a highly heterogeneous group of neuromuscular junction (NMJ) disorders clinically and genetically [1]. It compromises the safety margin required for reliable neuromuscular transmission [1]. Myasthenic symptoms, ranging from mild to severe and rapid worsening of weakness including an abrupt onset of respiratory failure, can be triggered by high-grade fever, infection, or agitation, particularly in CMS patients with paroxysmal apneas [2]. Diagnostic suspicions include early onset of the disease, easy fatigability or variable weakness, symmetrical involvement of orbital and limb muscles, as well as reduced responsiveness to repetitive nerve stimulation [3]. Typically, these disorders are caused by mutations affecting the postsynaptic components of NMJ and result from mutations that disturb the motor neuron endings. CMS includes a rare group that is being increasingly identified. Most of the newly recognized CMS-related genes cause presynaptic and synaptic NMJ defects [1,3].

SLC25A1 is a mitochondrial citrate transporter that intercedes the substitute of citrate/isocitrate with cytosolic malate [3]. Variants in the SLC25A1 gene are correlated with severe neurometabolic disorders such as combined D-2- and L-2-hydroxyglutaric aciduria (D/L-2-HGA). In this report, we present the case of a Saudi patient with CMS and a homozygous missense variant in SLC25A1. We conducted whole exome sequencing (WES) to determine the rudimentary causal gene [1,2]. The WES analysis revealed homozygous c.842C>T p.(Ala281Val) in the SLC25A1 gene [2]; however, it is noteworthy that more than 30 CMS-related genes have been discovered in recent years [1,3].

## Case presentation

**Figure 1:**
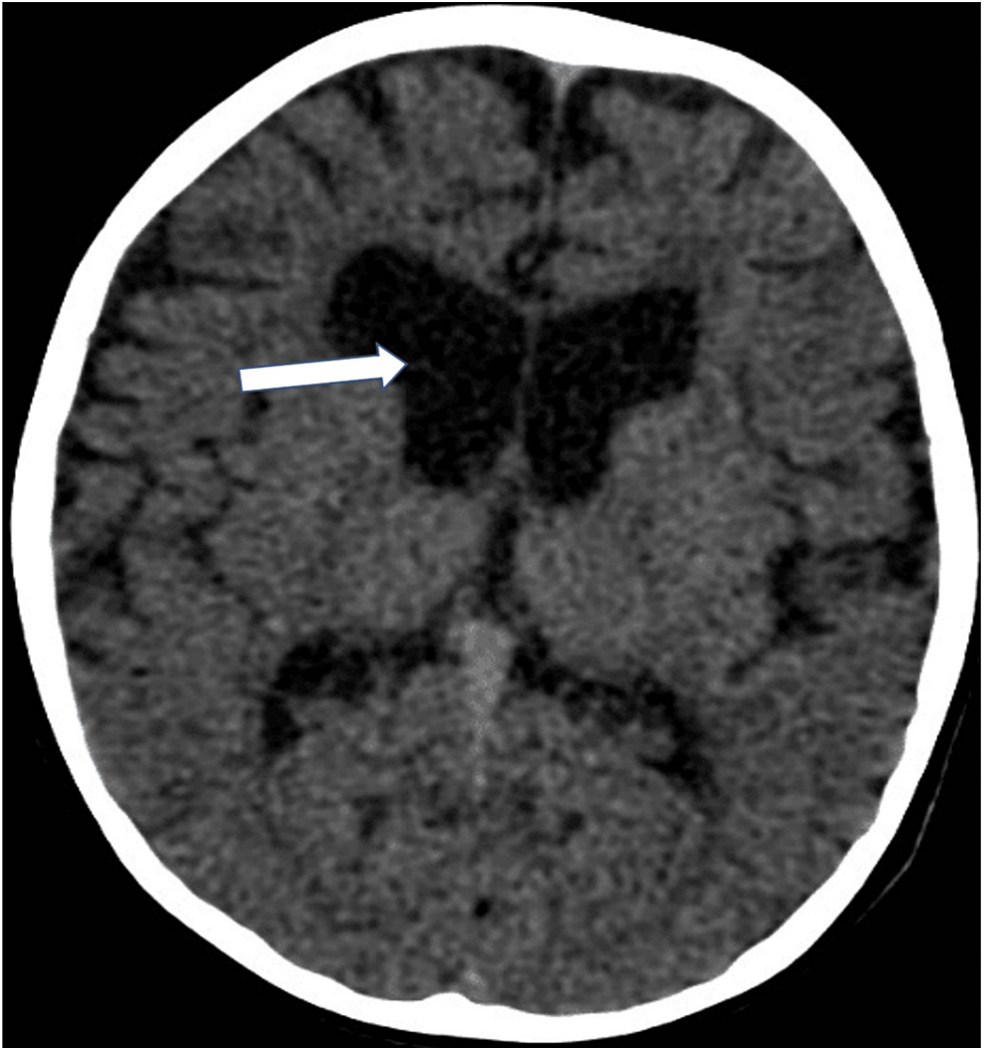
Non-enhanced CT brain axial section demonstrates dilated bilateral lateral ventricles CT: computed tomography

**Figure 2:**
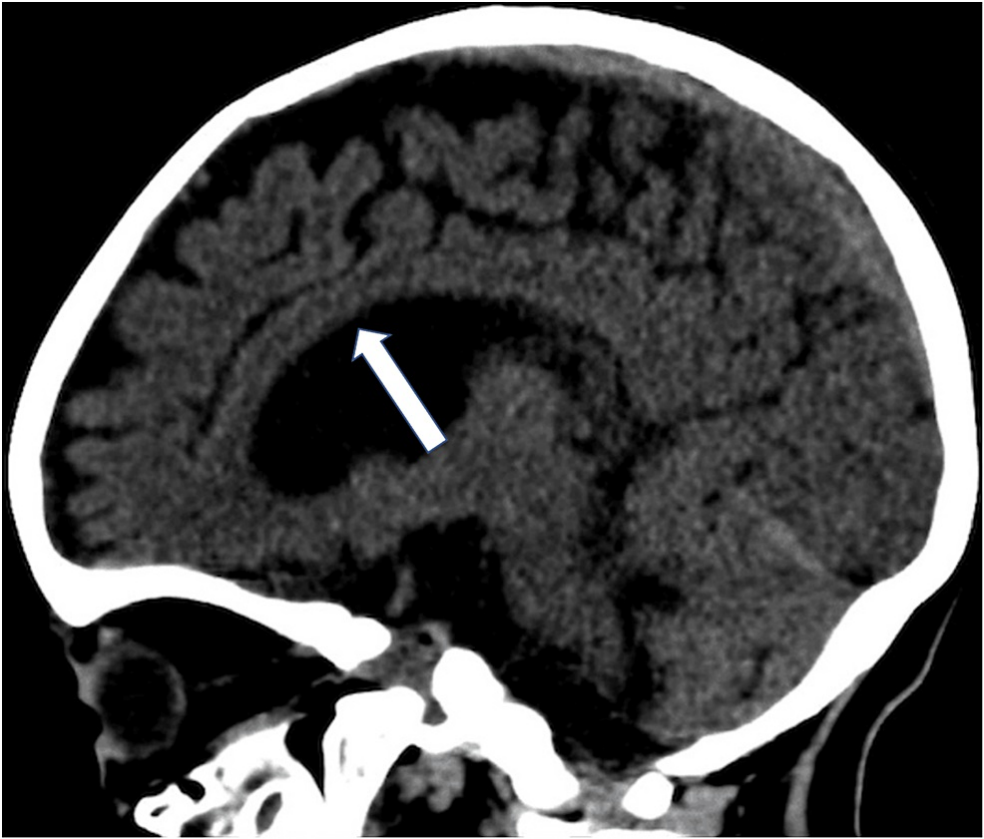
Non-enhanced CT brain sagittal section demonstrates corpus callosal thinning CT: computed tomography

The patient was a two-year-old full-term girl of consanguineous Saudi parents, a product of a cesarean section due to breech presentation, with a history of poor sucking, hypotonia, and bilateral ptosis, as well as recurrent pediatric intensive care unit (PICU) admission with apnea. Having an unremarkable family history, this girl's clinical journey had begun with the sudden cessation of breathing that had lasted more than 15 minutes, and she had then experienced arrest for three minutes. She had a history of choking and coughing. When the patient was examined, she was afebrile, dehydrated, and pale, with a Glasgow Coma Scale (GCS) score of 3. We began to stabilize her and she was intubated in the PICU where she received ketamine and intravenous midazolam, and was treated as a case of aspiration pneumonia. Therefore, she was given nebulized salbutamol, budesonide, and a regimen of antibiotics. A serial workup was conducted to reach a proper diagnosis. A genetic study and CT brain scan were conducted. During the follow-up in April 2022, the global developmental delay was noticed, and WES revealed a diagnosis of a homozygous, likely pathogenic, variant in the SLC25A1 gene. Additionally, the CT brain scan revealed volume loss of brain parenchyma associated with a mildly dilated ventricular system (Figure 1), extra-axial cerebrospinal fluid spaces, and thinning of the anterior part of the corpus callosum with the non-visualized posterior part (Figure 2). This finding is consistent with the genetic diagnosis of autosomal recessive combined D-2- and L-2-hydroxyglutaric aciduria, Fortunately, we suspected a CMS, and the initiation of management with pyridostigmine led to substantial improvement in the patient's condition.

## Discussion

We discussed the case of a patient from a Saudi family, who suffered from easy fatigability and weakness from even minimal movement, especially related to respiratory function (frequent apnea) [2,4]. The clinical phenotype was suggestive of a CMS [2,3]. CMS consists of a group of disorders of which 32 genes and clinical forms have been reported to date. A homozygous, likely pathogenic, variant was identified in the SLC25A1 gene, suggesting the diagnosis of CMS type 23 [Online Mendelian Inheritance in Man (OMIM) #618197] [2,4].

The SLC25A1 variant c.842C>T p.(Ala281Val) causes an amino acid change from Ala to Val at position 281 [1,4]. The substitution is close to the highly conserved donor splice site [2]. This variant has previously been described as disease-causing hydroxyglutaric aciduria. Combined D-2- and L-2-hydroxyglutaric aciduria (D/L-2-HGA) is an autosomal recessive neurometabolic disease expressed by severe muscular weakness, hypoxic-ischemic encephalopathy, intractable seizures, delay in the psychomotor development, and respiratory distress, and it results in early mortality. Imaging of the brain demonstrates abnormalities that include delayed myelination, ventricular dilatation, and cysts in the germinal layer (OMIM #615182). The SLC25A1 gene is located on chromosome 22q11, and the mitochondrial citrate carrier is encoded by SLC25A1 [2,4,5]. This protein corresponds to the mitochondrial carrier SLC25 family, which is mostly found in the mitochondrial inner membrane and is responsible for transporting a wide range of metabolites [5-6].

The disease phenotypes associated with SLC25A1 are thought to consist of a consecutive spectrum, including CMS, D/L-2-HGA, and intermediate types (with combined CMS and D/L-2-HGA characteristics) [4,6]. The presence of frequent apnea, early-onset fatigable weakness, and impaired NMJ transmission suggest the diagnosis of CMS. The lack of autoantibodies and therapeutic response also suggests a diagnosis of CMS [7]. 

This patient displayed significant fluctuating weakness, apnea, and a substantial pyridostigmine effect. As suggested by this patient's myasthenic crisis, clinicians should recognize ocular exacerbation or apnea as warning signs [6,7]. The results confirmed that SLC25A1 mutations may be associated with neuromuscular conduction impairment and the cause of CMS type 23, which includes characteristics varying from mild to mild with intellectual impairment. It is noteworthy that depletion of mitochondrial deoxyribonucleic acid (DNA) syndrome may manifest by progressive external ophthalmoplegia, which is defined as ptosis, restricted eye movement caused by immobility of the extraocular muscles, pharyngeal muscle weakness, and severe extremity weakness that can change and present as an inability to tolerate exertion [1,3,6].

In contrast, CMS is described as nonprogressive muscle weakness, a characteristic seen in all cases reported as CMS with SLC25A1 mutations [7]. To date, there have been less than 10 cases reported of this variant worldwide [8]. Our patient was the first case to be reported from Saudi Arabia. In terms of treatment, two other patients have shown functional benefits from treatment with acetylcholinesterase (AChE) inhibitors, whereas others did not benefit despite appropriate medication and dosing. One patient responded well to 3,4-diamino pyridine (DAP), consistent with presynaptic NMJ defects [8,9]. Patients with a mild phenotype and minimal symptoms may not require treatment with AChE inhibitors or 3,4-DAP [10]. However, it is worthwhile to try these drugs to assess and verify potential benefits, especially if lethargy and excessive fatigue are vital concerns [10].

In the present case, management with pyridostigmine resulted in substantial improvement in the patient's condition. We would like to emphasize that despite WES providing a molecular diagnosis, the results must be carefully interpreted. The diagnosis was made based on personal history, vital signs and symptoms, and positive response to pyridostigmine, while other diagnostic findings (electrophysiology and muscle biopsy) were unremarkable. Although a rare condition, CMS should be suspected in newborns with severe respiratory distress and hypotonia.

## Conclusions

We reported the case of a child with CMS that carried the SLC25A1 gene, and WES identified a likely pathogenic homozygous variant c.842C>T p.(Ala281Val), causing an amino acid change from Ala to Val at position 281. This finding is consistent with the genetic diagnosis of autosomal recessive combined D-2- and L-2-hydroxyglutaric aciduria, suggesting the diagnosis of CMS type 23 (OMIM #618197). We would like to emphasize that despite WES providing a molecular diagnosis, the results require careful interpretation and clinicians should recognize frequent apnea and respiratory failure triggered by infection or stress as warning signs of a myasthenic crisis. Further delineation of phenotypic variations requires more functional studies of SLC25A1.
